# Post-translational modifications as a key mechanism for herpes simplex virus type I evasion of host innate immunity

**DOI:** 10.3389/fmicb.2025.1543676

**Published:** 2025-02-11

**Authors:** Yongxing Zhang, Junlei Xie, Ying Feng, Abdul Qadeer, Shanni Li, Xu Deng, Lipeng Zhu, Bo Kong, Zanxian Xia

**Affiliations:** ^1^Department of Cell Biology, School of Life Sciences, Central South University, Changsha, China; ^2^Xiangya School of Pharmaceutical Sciences, Central South University, Changsha, China; ^3^School of Life Sciences, Xiangya School of Medicine, Central South University, Changsha, China; ^4^China Tobacco Hunan Industrial, Changsha, China; ^5^Hunan Key Laboratory of Animal Models for Human Diseases, Hunan Key Laboratory of Medical Genetics and Center for Medical Genetics, School of Life Sciences, Central South University, Changsha, China

**Keywords:** herpes simplex virus type 1, innate immunity, immune evasion, post-translational modifications, ubiquitination

## Abstract

Herpes simplex virus type 1 (HSV-1) is a DNA virus that infects humans and establishes long-term latency within the host. Throughout its prolonged interaction with the host, HSV-1 evades the innate immune system by encoding its own proteins. Post-translational modifications (PTMs) of these proteins play crucial roles in their function, activity, and interactions with other factors by modifying specific amino acids, thereby enabling a diverse range of protein functions. This review explores the mechanisms and roles of PTMs in HSV-1-encoded proteins, such as phosphorylation, ubiquitination, deamidation, and SUMOylation, during HSV-1 infection and latency. These modifications are essential for suppressing host innate immunity, facilitating viral replication, and elucidating the crosstalk among various post-translational modifications.

## 1 Introduction

More than 100 types of herpesviruses have been identified to date, categorized into three subfamilies: alpha, beta, and gamma herpesviruses (Sehrawat et al., 2018). HSV-1 is globally prevalent due to its high infectious potential and ability to establish latency (James et al., 2020). HSV-1 belongs to the alpha-herpesvirus subfamily based on its genome, viral characteristics, and incubation period. It is a double-stranded DNA virus with a core genome of approximately 152 kb, encoding more than 80 genes. These genes translate into the capsid proteins that encase the DNA core, membrane proteins that form the protein matrix, and the outer envelope proteins and glycoproteins. These viral proteins play essential roles throughout the HSV-1 life cycle, encompassing processes such as assembly, infection, replication, and latency (Su et al., 2024).

Proteins are the primary executors of biological activities, and their functions are often regulated by post-translational modifications (PTMs) (Lin et al., 2015). PTMs involve the addition of chemical groups, peptides, or other proteins to specific residues of protein through distinct enzymatic reactions following protein synthesis. This modification enhances protein versatility and diversity by regulating protein-protein interactions, enzyme activity, and gene expression. Over 400 known types of PTMs have been identified, including methylation, acetylation, ubiquitination, and phosphorylation (Ebert et al., 2022; Zhong et al., 2023).

Viruses, including HSV-1, exploit host cell metabolism to complete their life cycle. Regulating PTMs in host cells is a key strategy viruses employ to enhance their infection and replication (Cheng et al., 2022). Over millennia of co-evolution within the human immune system, HSV-1 has refined its ability to manipulate PTMs, including phosphorylation, methylation, and glycosylation, to optimize infection and evade the host’s innate immune response. Additionally, host proteins can inhibit viral replication by affecting the PTMs of viral proteins, such as degrading viral proteins and suppressing the activity of associated proteins (Kulej et al., 2017). In this review, we examine the role of PTMs in HSV-1 encoded protein, focusing on phosphorylation, ubiquitination, glycosylation, and SUMOylation, and their role in suppressing the host’s innate immune pathways. We also explore the crosstalk between different PTMs during the HSV-1 life cycle and their contribution to the virus’s ability to persist in the host and establish a lifelong latent infection.

## 2 HSV-1 infection and host innate immune response

HSV-1 infection typically occurs during childhood, primarily affecting the mucosal epithelial cells (Yousuf et al., 2020). This infection can lead to a range of symptoms, including oral or facial herpes, and may also involve the genitals or other areas of the body. The incubation period generally spans from 1 to 26 days, with common symptoms manifesting as small blisters at the corners of the mouth or around the nostrils, accompanied by pain and tingling (Gupta et al., 2007). HSV-1 follows a distinct infection pattern that includes primary, latent, and recurrent infections. During the primary infection, the virus invades epithelial cells and replicates in the host cell nucleus. Mature virions are released through exocytosis, allowing the infection to spread to neighboring cells (Danastas et al., 2020; Nicola et al., 2005). Once the virus enters sensory neurons, it is transported retrogradely along microtubules to sensory ganglia, such as the trigeminal ganglia, with the assistance of key proteins pUL36 and pUL37, thereby establishing a latent infection (Kim et al., 2024; Richards et al., 2017). Various stressors can trigger the reactivation of latent HSV-1, resulting in renewed replication and recurrent infections (Yan et al., 2020). The cycle of primary infection, latency, and reactivation constitutes the complete life cycle of HSV-1 infection (Marcocci et al., 2020). Notably, HSV-1 exhibits a strong neuro-infective nature, and emerging evidence suggests a link between its infection and the onset and progression of neurodegenerative diseases, such as Alzheimer’s disease (AD) (Mancuso et al., 2019; Sivasubramanian et al., 2022).

The host’s innate immune response serves as the primary mechanism against viral infections (Kawai and Akira, 2006; Koyama et al., 2008). Upon HSV-1 infection, host cells recognize viral pathogen-associated molecular patterns (PAMPs) through pattern recognition receptors (PRRs). The most common viral recognition receptors in host cells include toll-like receptors (TLRs) and Retinoic acid-inducible gene-I (RIG-I)-like receptors (RLRs) (Alandijany, 2019). TLRs are generally categorized as either cell surface receptors and endosomal receptors based on their subcellular localization (Ma and He, 2014; Zolini et al., 2014). Upon viral infection, TLRs activate immune signaling pathways to suppress viral replication (Bryant-Hudson et al., 2013; Kim, 2023). RLRs are immune receptors that detect viral RNA, with key members such as RIG-I, melanoma differentiation-associated gene 5 (MDA5), and laboratory of genetics and physiology 2 (LGP2) (Loo and Gale, 2011). After binding to viral RNA, these receptors activate signaling pathways that engage the upstream regulator mitochondrial antiviral signaling protein (MAVS), which then activate transcription factors such as interferon regulatory factor 3 (IRF3) and nuclear factor kappa-light-chain-enhancer of activated B cells (NF-κB) (Seth et al., 2005). This cascade ultimately stimulates the production of interferons and inflammatory cytokines, which help the immune system clear the virus (Rehwinkel and Gack, 2020).

During HSV-1 infection, TLRs and RLRs detect double-stranded RNA (dsRNA) intermediates, initiating the interferon response (Zhu and Zheng, 2020). Additionally, stimulators of interferon genes (STING) and NOD-like receptors (NLRs) contribute to inhibiting viral replication and transmission through similar mechanisms (Liwinski et al., 2020). Protein kinase R (PKR) is another critical sensor of viral RNA, and its activation triggers an antiviral response that inhibits viral replication. PKR plays a crucial role in inflammation and immune dysfunction by regulating several key pathways, including mitogen-activated protein kinases, IRF3, NF-κB, apoptosis, and autophagy (Kang and Tang, 2012). Studies have shown that HSV-1 has evolved strategies to manipulate PKR and evade its antiviral effects (Pennisi et al., 2020; Pennisi and Sciortino, 2023).

Additionally, DNA recognition molecules, such as DNA sensors, primarily function by identifying specific regions of single-stranded or double-stranded DNA, including AT-rich sequences or phosphorylated modified domains, such as organophosphates (Zahid et al., 2020). When a DNA sensor detects a non-self DNA structure or sequence, it activates signaling pathways, such as the cyclic GMP–AMP synthase-stimulator of interferon genes (cGAS-STING) pathway or the absent in melanoma 2 (AIM2) pathway, which stimulate the host immune system and initiate an immune response (Chen et al., 2016; Xu et al., 2023). These pathways are associated with cytoplasmic DNA sensors that are prevalent across various cell types. For instance, during the HSV-1 replication and assembly, the double-stranded DNA (dsDNA) released into the cytoplasm is recognized and processed by cGAS. The activated cGAS then binds to ATP and GTP to produce cyclic GMP-AMP (cGAMP), which triggers an antiviral immune response by activating STING and downstream signaling adapters (Hopfner and Hornung, 2020; Zhang et al., 2020). Additionally, Interferon Gamma Inducible Protein 16 (IFI16), a recently identified DNA sensor, can recognize both its aberrant dsDNA and viral dsDNA, leading to the production of type I interferon (IFN-I) and contributing to inflammatory responses (Briard et al., 2020; Unterholzner et al., 2010).

**FIGURE 1:**
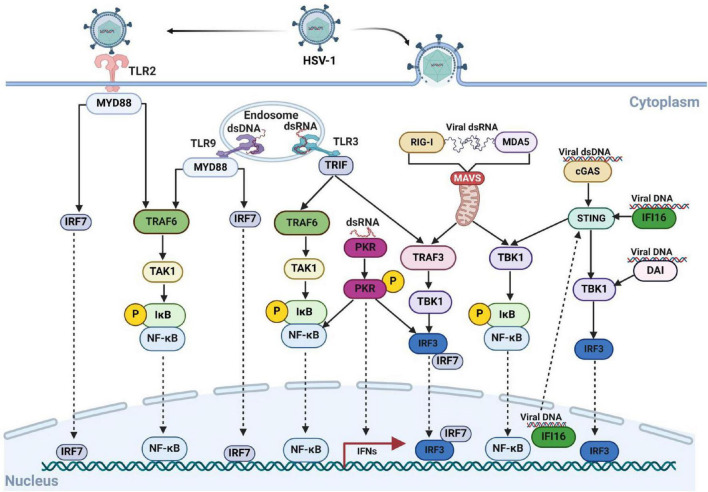
The innate immune response is activates by the entry of HSV-1 into host cells. HSV-1 is recognized by PRRs, which trigger a signaling cascade that leads to the production of IFNs and cytokines. The entry of HSV-1 into the cell involves both the TLR and non-TLR pathways. Key viral DNA sensors include TLR9, cGAS, IFI16, and DAI, while viral RNA sensors include TLR3, MDA5, PKR and RIG-I. During viral attachment and entry, multiple virus-recognizing receptors detect viral components and initiate innate immune responses. Created with BioRender.com.

Activation and negative regulation of various signaling pathways are often closely associated with PTMs of key factors, especially innate immune pathways (Diskin et al., 2021). For example, NF-κB is a crucial transcription factor that regulates antiviral immune responses and the expression of interferons (Li and Verma, 2002). A key step in NF-κB activation is the phosphorylation of inhibitor of kappa B alpha (IκBα) by IKK kinase, which leads to the degradation of IκBα via the ubiquitin-proteasome pathway and the release of NF-κB into the nucleus (Hayden and Ghosh, 2011). Similarly, the interferon alpha/beta receptor-Janus kinase-signal transducer and activator of transcription (IFNAR-JAK-STAT) signaling pathway is a vital component of the immune system, playing a central role in regulating immune cell function and antiviral as well as antitumor responses (Darnell et al., 1994). Upon binding to IFNAR, interferon-α/β activates JAK, which phosphorylates tyrosine residues on IFNAR, facilitating the recruitment of STAT proteins, which are subsequently phosphorylated. The phosphorylated STAT proteins form dimers that translocate to the nucleus, where they initiates the transcription and translation of target genes. This process leads to the production of numerous antiviral interferon-stimulated genes (ISGs) proteins (Raftery and Stevenson, 2017; Wang et al., 2021). We have graphically illustrate the host-associated innate immune response after HVS-infection, as shown in Figure 1.

## 3 Post-translational modifications in HSV-1 infection

In recent years, advancements in mass spectrometry have significantly enhanced our understanding of the molecular mechanisms underlying virus-host interactions (Ashcroft, 2019). This technology has provided valuable insights into how viruses regulate important critical cellular processes such as cell cycle, transcription, translation, and the degradation of antiviral proteins, through PTMs (Bai et al., 2021). These regulatory mechanisms are important in influencing both latent and recurrent infection mechanisms (Milewska et al., 2020).

HSV-1 virus has evolved sophisticated strategies to establish both acute, latent, and recurrent infections in human hosts. Over thousands of years, it has effectively evaded the host’s antiviral innate immune responses (Wang et al., 2024). Among these strategies, PTMs serve as a key regulatory mechanism during HSV-1 infection. The virus leverages PTMs to regulate its gene expression and protein function, targeting viral proteins for modification or using them as competitors to enhance its infectivity and resistance to host innate immune responses, as shown in Table 1 (Kumar et al., 2020).

**TABLE 1:** PTMs in key viral proteins of HSV-1 involved in immune function.

PTMs	Viral protein	Targets	Function	References
Phosphorylation	US3	TSC2	Inhibit mTORC1 in an Akt-independent manner	Chuluunbaatar et al., 2010
		ULK1	Reducing ULK1 activity, inhibits host autophagy	Rubio and Mohr, 2019
		Beclin	Inhibits Beclin1-dependent autophagy	Rubio and Mohr, 2019
		β-catenin	Blocking β-catenins nuclear translocation	You et al., 2020
		RelA	Hyperphosphorylated RelA and block its nuclear translocation	Wang et al., 2014
		IRF3	Hyperphosphorylated IRF3 to prevent IRF3 activation	Wang et al., 2013b
		RIG-1	Prevents the binding of RIG-I to MAVS	Van Gent et al., 2022
		Viral VHS	Inhibiting the accumulation of phosphorylated PKR	Pennisi et al., 2020
	ICP34.5	NOP53	Promote the dephosphorylation of eIF2α	Meng et al., 2018
	UL13		Inhibiting the accumulation of phosphorylated PKR	Pennisi et al., 2020
	VP22	Viral ICP0	Affect ICP0 expression and localization	Potel and Elliott, 2005
	VP11/12	Dok-2	Facilitating Dok-2 degradation	Lahmidi et al., 2017
	ICP6	RIP1/RIP3	Lead to necrosis in mouse cells, inhibits TNF-induced necrosis in human cells	Huang et al., 2015
Ubiquitination	ICP0	PML, Sp100	Affects the establishment of ND10	Everett et al., 2008
		RNF8, RNF168	Inhibits the DNA damage pathway mediated by RNF8 and RNF168	Lilley et al., 2010
		MyD88, Mal	Reducing the response of NF-κB signaling pathway	Van Lint et al., 2010
		DNA-PKcs	Interferes the host DNA damage repair function	Parkinson et al., 1999
		BRCC36	Downregulates the expression of IFNAR1	Zhang et al., 2021
		SLFN5	Inhibiting SLFN5’s antiviral effects	Kim et al., 2021
		IFI16	Suppress the function of DNA sensor	Diner et al., 2015
		MORC3	Suppression of MORC3-regulated DNA elements	Gaidt et al., 2021
		IRF7	Affects the expression of IRF7	Shahnazaryan et al., 2020
		p50	Inhibiting NF-κB-dependent gene expression	Zhang et al., 2013
	UL21	TOLLIP	Downstream TBK1 and IRF3 signaling pathways	Ma et al., 2023
	US3	LAT	Suppresses TCR signaling and T-cell activation	Yang et al., 2015
		Bclaf1	Reducing IFN-induced antiviral activity	Qin et al., 2019
Deubiquitination	UL36	IκBα	Inhibiting cGAS induced IFN-β and NF-κB promoter activation	Ye et al., 2017
		TRAF3	Prevent the recruitment of TBK1	Wang et al., 2013a
		IFNAR2	Antagonize the IFN-JAK-STAT signaling pathway	Yuan et al., 2018
		TSG101	Inhibits inflammasome formation and antigen presentation	Kharkwal et al., 2016
Deamidation	UL37	cGAS	Resulting in the loss of cGAMP synthesis	Zhang et al., 2018
		RIG-I	Inhibits the recognition of HSV-1 by RIG-I	Huang et al., 2021
SUMOylation	ICP27	Daxx	Suppresses p65 acetylation and NF-κB signaling	Christensen et al., 2016
	ICP0	PIAS1	Disrupt the interaction between PIAS1 and PM	Shih et al., 2007

### 3.1 Phosphorylation in HSV-1 infection

Phosphorylation is a process in which certain amino acid residues in proteins form covalent bonds with phosphate groups derived from ATP. This modification mainly takes place on serine (Ser), threonine (Thr), and tyrosine (Tyr). Additionally, phosphorylation has been reported on histidine (His), arginine (Arg), lysine (Lys), aspartic acid (Asp), glutamic acid (Glu), and cysteine (Cys) as well (Watanabe and Osada, 2012).

#### 3.1.1 HSV-1 Us3 protein as a kinase-mediated modifier of host proteins

HSV-1 encodes Us3, a highly conserved serine/threonine kinase within the Herpesvirus alpha subfamily, which plays a critical role in inhibiting host interferon expression. The kinase activity of Us3 depends on phosphokinase active sites, K220 and D305 (Mori, 2012). It has been reported that Us3 activates mTORC1 in infected cells in a protein kinase B (Akt)-independent manner by directly phosphorylating Tuberous Sclerosis Complex 2 (TSC2) following HSV-1 infection (Chuluunbaatar et al., 2010). Moreover, Us3 inhibits host autophagy by phosphorylating Unc-51 ike autophagy activating kinase 1 (ULK1), a major autophagy activator, via mTORC1, thereby reducing ULK1 activity. Us3 also functions as an Akt-like kinase, interacting with the host protein Beclin1 and phosphorylating it at S295 and S234 sites. This inhibits Beclin1-dependent autophagy by hijacking Akt signaling (Rubio and Mohr, 2019). Furthermore, HSV-1 Us3 interacts with β-catenin and mediates its hyperphosphorylation at Thr556, thereby blocking its nuclear translocation and inhibiting IFN-I production (You et al., 2020). Us3 further induces the hyperphosphorylation of IRF3 and RelA/p65, which inhibits the IRF3 and NF-κB signaling pathways and suppress interferon production (Wang et al., 2014; Wang et al., 2013b).

**FIGURE 2:**
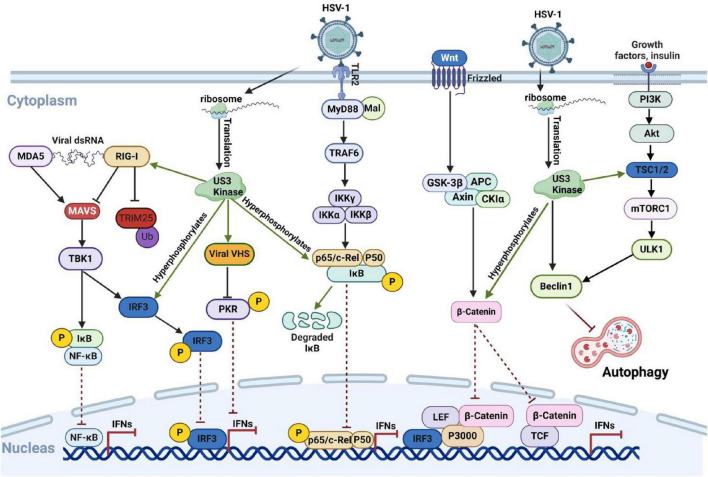
HSV-1 Us3 protein inhibits the host immune response by phosphorylating host proteins via its kinase activity. Following HSV-1 infection, host cells detect the viral nucleic acid sequences through various pathways, triggering the intrinsic antiviral immune response to combat viral proliferation and eliminate infected cells. The Us3 protein encoded by HSV-1 disrupts signal transmission in these pathways by phosphorylating key intermediate proteins, including IRF3 and RelA, thereby suppressing the host’s antiviral response. Created with BioRender.com.

A recent study indicates that HSV-1 Us3 interacts with RIG-I and specifically phosphorylates its S8 residue. This phosphorylation inhibits TRIM25-mediated ubiquitination of RIG-I, prevents RIG-I binding to MAVS, and ultimately reduces of type I interferon induction (Gack et al., 2007; Van Gent et al., 2022). Moreover, Us3 play a vital role in preventing the accumulation of the phosphorylated PKR. Us3 interacts with the viral protein VHS, mediating VHS phosphorylation, which regulates PKR-mediated immune responses (Pennisi et al., 2020). Taken together, Us3 phosphorylates several key factors in the host immune pathway. These phosphorylation events benefit viral growth and replication, as illustrated in Figure 2. Therefore, Us3 may serve as a critical target for designing HSV-1 inhibitors or vaccines.

#### 3.1.2 Phosphorylation of HSV-1 protein interferes with host innate immunity

The HSV-1-encoded protein ICP34.5 plays a critical role in interfering with or disrupting several antiviral pathways at multiple levels (Ripa et al., 2022). Studies have shown that the ectopic expression of the nucleolar protein NOP53 significantly increases both intracellular and extracellular HSV-1 viral yields in type I interferon-deficient Vero cells. However, this effect is absent in mutant viruses lacking ICP34.5. Further research reveals that ICP34.5 utilizes NOP53 to promote the dephosphorylation of the eukaryotic initiation factor 2 (eIF2α), thereby enabling efficient viral translation (Meng et al., 2018). HSV-1 UL51 is a phosphoprotein critical for viral envelope formation and cell-to-cell spread, enhancing the replication efficiency in cell cultures (Roller et al., 2014). Phosphorylation at serine 184 (Ser-184) of UL51 significantly reduces viral replication and cell spread in HaCaT cells, as demonstrated by mass spectrometry analysis. Additionally, alanine mutation at UL51 Ser-184 significantly decrease mortality rate in mice following ocular infection (Kato et al., 2018). Furthermore, UL13, along with VHS and US3, has been shown to inhibit PKR phosphorylation, highlighting its role in disrupting antiviral immune responses (Pennisi et al., 2020).

VP22, an envelope protein of HSV-1, undergoes extensive phosphorylation during infection (Chi and Blaho, 2024). Notably, ICP0, encoded by an immediate early gene of HSV-1, exhibits E3 ubiquitin ligase activity that facilitates the degradation of IRF3, a critical component of the antiviral response (Lin et al., 2004). This degradation necessitates the cytoplasmic localization of ICP0, which is influenced by the phosphorylation states of VP22. Non-phosphorylated VP22 inhibits ICP0 expression, while permanently phosphorylated VP22 diminishes ICP0 packaging (Potel and Elliott, 2005). In addition, the HSV-1 envelope protein VP11/12 regulates T cell signaling pathways by activating the Src family tyrosine kinases (SFK), which enhance T cell responses. Mutations in VP11/12 impair SFK activation and downstream of tyrosine kinase 2 (Dok-2) phosphorylation, disrupting the maintenance of CD8+ T cells. This mechanism also influences the negative feedback loop following antigen clearance by facilitating Dok-2 degradation, which terminates T cell receptor (TCR) signaling and promotes the resting state of T cells (Lahmidi et al., 2017). Necrosis is a form of programmed cell death mediated by signaling complexes containing the receptor-interacting protein 3 (RIP3) and RIP1 kinase. A study has shown that the interaction between ICP6, encoded by HSV-1, and RIP1/RIP3 produces nearly opposite effects in mouse and human cells. Specifically, heterodimeric interactions of RIP1 and RIP3, as well as homodimeric interactions of RIP3, lead to necrosis in HSV-1-infected mouse cells. In contrast, the RHIM structural domain of HSV-1 ICP6 inhibits TNF-induced necrosis in human cells (Huang et al., 2015; Mocarski et al., 2015).

#### 3.1.3 HSV-1 interferes with normal host phosphorylation to evade innate immunity

HSV-1 encodes multiple proteins that disrupt normal host phosphorylation mechanisms to evade the innate immune response. One such protein, UL2, antagonizes Tumor Necrosis Factor Alpha (TNF-α)-mediated NF-κB activation. UL2 interacts with NF-κB subunits p65 and p50 without affecting the formation of p65/p50 dimers or their nuclear localization. Instead, UL2 inhibits NF-κB activity by attenuating TNF-α-induced phosphorylation at the p65 Ser536 locus, reducing the expression of downstream inflammatory chemokines such as Interleukin 8 (IL-8) (Cai et al., 2020). The HSV-1 immediate early protein ICP22 inhibits the phosphorylation of Ser2 in the carboxy-terminal domain (CTD) of RNA polymerase II (pol II), disrupting its productive elongation. Co-immunoprecipitation (Co-IP) analysis and mass spectrometry have identified transcriptional elongation factors, including P-TEFb, various CTD kinases, and Facilitates Chromatin Transcription (FACT) complexes, as key players of ICP22 in human cells (Isa et al., 2021). Further studies demonstrated that ICP22 interacts with cyclin-dependent kinase 9 (CDK9) and other CDKs, thereby inhibiting the transcriptional elongation of cellular genes—a mechanism potentially critical to HSV-1 pathogenesis (Whisnant et al., 2024). The HSV-1 early protein ICP27 plays a crucial role in regulating viral gene expression and is associated with viral latency and reactivation. ICP27 binds to IκBα, inhibiting its phosphorylation and subsequent ubiquitin-mediated degradation. This inhibition prevents IκBα from dissociating with NF-κB dimers, thereby suppressing NF-κB signaling pathway activation (Hargett et al., 2006; Kim et al., 2008). Additionally, the early protein ICP34.5 and the kinase US11 work together to counteract the host’s translation shutdown during viral replication by inhibiting eIF2 phosphorylation in the later stages of HSV-1 infection (Charron et al., 2019; Lussignol et al., 2013). In summary, HSV-1 disrupts normal host phosphorylation processes to evade innate immunity.

### 3.2 Ubiquitination in HSV-1 infection

Ubiquitination is a three-step enzymatic process involving the ubiquitin-activating enzyme E1, the ubiquitin-conjugating enzyme E2, and the ubiquitin ligase E3. In this process, E1 activates ubiquitin molecules in the presence of ATP and transfers them to E2. The ubiquitin ligase E3 then facilitates the attachment of ubiquitin from E2 to the target protein. Once ubiquitination occurs, most ubiquitin-tagged proteins are recognized and degraded by the 26S proteasome. Notably, ubiquitination is reversible, as deubiquitinases (DUBs) can hydrolyze bonds at the carboxyl terminus of ubiquitin, thereby reversing protein degradation and modulating protein function (Liu et al., 2024).

#### 3.2.1 HSV-1 proteins as E3 ligases or deubiquitinating enzymes in HSV-1 infection

The immediate-early protein ICP0, encoded by HSV-1, is a multifunctional protein with E3 ubiquitin ligase activity that is critical for HSV-1 replication and immune evasion (Everett, 2000). The N-terminal finger-ring structure (RING finger) of ICP0 confers E3 ubiquitin ligase activity, enabling it to ubiquitinate multiple host proteins and disrupt the host’s antiviral defenses (Boutell and Everett, 2013). ICP0 primarily enhances HSV-1 gene expression and replication by counteracting host restriction factors, such as interferon responses (Everett and Orr, 2009), DNA damage responses (Lilley et al., 2011), and chromatin repression (Gu and Zheng, 2016).

When HSV-1 invades host cells, the innate immune system restricts the viral genome access to the nuclear site by targeting PML nuclear bodies (PML-NBs), also known as nuclear domain 10 (ND10). This restriction promotes the transcriptional silencing of viral genes and prevents viral lytic replication (Jan Fada et al., 2023; Wang et al., 2012). After HSV-1 enters the cell, ICP0 utilizes its nuclear localization signal and ND10 localization domain to accurately localize to the ND10 complex in the nucleus. It employs its E3 ligase property to degrade the PML directly or indirectly and Sp100 proteins, which in turn affects the establishment of Nuclear Domain 10 (ND10) and its inhibitory effect on HSV-1 infection (Everett et al., 2008; Ma et al., 2022; Xu et al., 2016). HSV-1 further disrupts the host DNA damage response. For example, ICP0 interferes with the host DNA damage repair function by degrading DNA-Dependent Protein Kinase Catalytic Subunit (DNA-PKcs) through ubiquitination, thereby increasing the viral replication multiplier (Parkinson et al., 1999). Additionally, it inhibits the DNA damage pathway mediated by RNF8 and RNF168 as E3 ligases through ubiquitination (Lilley et al., 2010).

ICP0 also modulates immune signaling pathways. It inhibits STAT1 activation, affecting antivirus signaling (Halford et al., 2006), and blocks IRF3- and IRF7-mediated activation of interferon-stimulated genes (Lin et al., 2004; Paladino et al., 2010), with ICP0 specifically promoting IRF7 ubiquitination (Shahnazaryan et al., 2020). Additionally, ICP0 degrades MORC3, suppressing MORC3-regulated DNA elements (MREs) near the IFNB1 locus, which are critical for interferon expression (Gaidt et al., 2021; Sloan et al., 2016). In IFI16-deficient cells, replication of ICP0-mutant HSV-1 increases, highlighting ICP0’s role in IFI16 degradation (Diner et al., 2015; Orzalli et al., 2012). Furthermore, ICP0 reduces protein levels of Myeloid Differentiation Primary Response Gene 88 (MyD88) and MyD88 Adaptor-Like (Mal), limiting the inflammatory response of the TLR2-stimulated NF-κB signaling pathway following HSV-1 invasion (Van Lint et al., 2010). By binding to the NF-κB subunits p65 and p50, ICP0 inhibits NF-κB-dependent gene expression through p50 degradation, but not p65 (Zhang et al., 2013). ICP0 also induces K48-linked polyubiquitination and BRCA1-dependent protein complex 36 (BRCC36) degradation, which downregulates IFN-I receptor interferon alpha/beta receptor 1 (IFNAR1) and impairs the host’s IFN-I antiviral response during early HSV-1 infection (Zhang et al., 2021). More recently, a comparative proteomics study found that Schlafen5 (SLFN5), an HSV-1 inhibitory factor that inhibits viral transcription, is also regulated by ICP0. During HSV-1 infection, SLFN5 binds to Viral DNA and is targeted for ubiquitin-mediated degradation by ICP0, inhibiting SLFN5’s antiviral effects (Kim et al., 2021).

**FIGURE 3:**
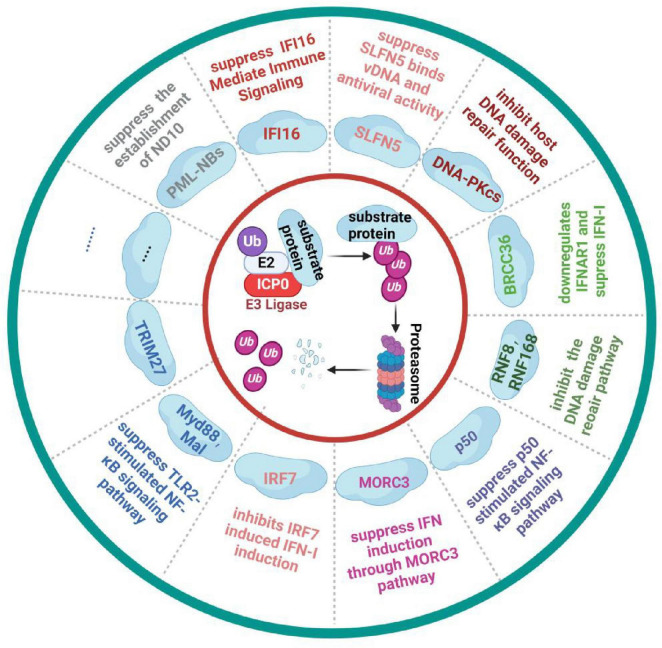
HSV-1 ICP0 protein inhibits the host immune response by degrading host proteins through the ubiquitin-proteasome pathway. Upon entering the host cells, HSV-1 uses the host’s cellular machinery to synthesize the key immediate-early protein ICP0. Through its E3 ligase activity, ICP0 mediates the ubiquitin-dependent degradation of key immune pathway protein, such as IFI16 and MOCR3, effectively suppressing the host’s antiviral response. Figure created with BioRender.com.

In summary, ICP0 targets numerous ubiquitination substrates using its RING finger structure, as illustrated in Figure 3. The development of mass spectrometry technology offers opportunities to discover additional critical substrate proteins. As an essential multifunctional protein encoded by HSV-1, ICP0 represents a promising target for developing HSV-1-specific drugs and inhibitors.

Moreover, ICP0 is regulated by both viral and host proteins. For instance, HSV-1 ICP134.5 interacts with ICP0, preventing its proteasomal degradation (Manivanh et al., 2017). Conversely, the host E3 ligase tripartite motif containing 23 (TRIM23) targets ICP0 for degradation by inducing K11-and K48-linked ubiquitination (Liu et al., 2021).

UL36, the largest envelope protein in the herpesvirus family and a well-conserved protein, contains a DUB motif at its N-terminus, granting it deubiquitinase activity (Newcomb and Brown, 2010). An early study revealed that UL36 suppresses NF-κB promoter activation and IFN-β production through DNA sensors. UL36 deubiquitinates IκBα, limiting its degradation and thereby inhibiting cGAS induced IFN-β production and NF-κB activation (Ye et al., 2017). UL36 also interacts with TNF receptor-associated factor 3 (TRAF3) and deubiquitinates it to prevent the recruitment of the downstream adapter TANK-binding kinase 1 (TBK1) (Wang et al., 2013a). Additionally, another study has demonstrated that during the ectopic expression of UL36 and wild-type HSV-1 infection, UL36 specifically binds to IFNAR2 and inhibits its interaction with JAK1, thereby antagonizing the IFN-JAK-STAT signaling pathway (Yuan et al., 2018). UL36 has also been reported to affect the endosomal sorting complex required for transport (ESCRT)-I core component tumor susceptibility gene 101 (TSG101). UL36 removes the ubiquitin tag from TSG101, interacts with it, and prevents its degradation by host cells. This disruption alters the intracellular and extracellular distribution of TSG101, affects the formation of the ESCRT-I complex, and inhibits inflammasome formation and antigen presentation, allowing HSV-1 to evade host immune surveillance (Kharkwal et al., 2016).

#### 3.2.2 HSV-1 proteins modulate host protein ubiquitination for immune evasion

During HSV-1 infection, the viral protein UL21 bridges the E3 ligase ubiquitin-protein ligase E3 Component C (UBE3C) and cGAS, triggering the K27-linked ubiquitination and degradation of cGAS at its K384 residue. UL21 interacts with the selective autophagy receptor Toll-interacting protein (TOLLIP), recognizes the ubiquitinated cGAS, and directs it to the lysosome for degradation. This process inhibits the downstream TBK1 and IRF3 signaling pathways, thereby reducing the production of type I interferon production (Le Sage et al., 2013; Ma et al., 2023). Additionally, Us3 disrupts T-cell signaling by inhibiting TRAF6-mediated LAT ubiquitination, which is crucial for LAT tyrosine phosphorylation and T-cell activation. This interruption suppresses TCR signaling and T-cell activation (Yang et al., 2015). Us3 also induces the degradation of host protein BCL2-associated transcription factor 1 (Bclaf1) through the ubiquitin-proteasome pathway, significantly reducing IFN-induced antiviral activity (Qin et al., 2019).

### 3.3 Glycosylation during HSV-1 infection

HSV-1 glycoprotein B (gB) is a class III fusion glycoprotein and a key target for antibody-mediated immunity (Ramachandran et al., 2010). Glycosylation at the Asn 141 site of gB structurally conceals essential amino acids involved in antibody recognition and viral fusion. This creates a glycan shield that blocks antibody binding to gB epitopes, allowing the virus to evade immune surveillance. For instance, while non-glycosylated gB can mediate antibody-dependent cellular cytotoxicity (ADCC), glycosylation of Asn141 significantly inhibits the ADCC response (Fukui et al., 2023; Jambunathan et al., 2021).

HSV-1 gC-1 is a glycoprotein composed of 511 amino acid residues and is extensively glycosylated, with nine consensus sites for N-linked glycosylation and a prominent mucin-like domain. This domain, spanning amino acids 30 to 124, is rich in Ser and Thr residues, which serve as sites for O-linked glycosylation. The O-linked glycan structures on gC-1 shield viral envelope glycoproteins from potential neutralizing antibodies, thereby facilitating immune evasion (Harris et al., 1990; Komala Sari et al., 2020).

### 3.4 Deamidation during HSV-1 infection

**FIGURE 4:**
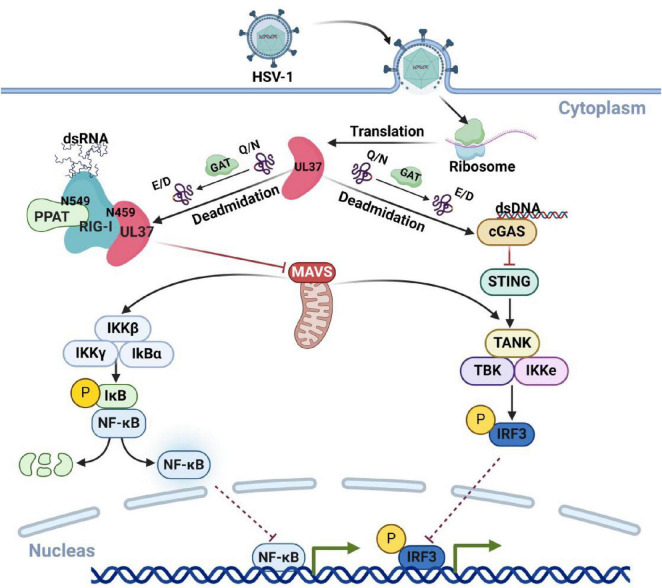
HSV-1 UL37 protein deamidates host proteins to inhibit immune pathways. UL37, a capsid protein encoded by HSV-1 with deamidase activity, inhibits host antiviral immune responses by targeting key viral recognition sensors. Within host cells, UL37 deamidates cGAS and RIG-I, preventing the recognition of HSV-1 viral nucleic acids and effectively suppressing host-associated antiviral immune responses. Created with BioRender.com.

UL37, a capsid protein encoded by HSV-1, has been identified as a deamidase. UL37 binds to RIG-I and mediates deamidation at the N495 site. This modification inhibits RIG-I recognition of HSV-1 and suppresses the host’s intrinsic immunity (Zhao et al., 2016). Moreover, the deamidation of RIG-I at N495 by UL37 facilitates its re-amidation at the N549 site by the host deamidase PPAT (Huang et al., 2021). The synergistic deamidation of RIG-I by UL37 and PPAT allows HSV-1 to evade RIG-I recognition. Beyond RIG-1, UL37 has also been shown to inhibit the activation of cGAS through its deamidase activity, resulting in the loss of cGAMP synthesis and the blockage of downstream signaling. Notably, HSV-1-infected cells with a mutation that eliminates UL37’s deamidase activity do not exhibit this inhibition (Zhang et al., 2018). These findings highlights UL37’s critical role as a deamidase in HSV-1’s evasion of host immunity, as shown as in Figure 4.

### 3.5 SUMOylation in HSV-1 infection

HSV-1 ICP27 is a multifunctional protein essential for viral replication, late gene expression, and reactivation from latency (Sandri-Goldin, 2011). ICP27 also regulates the cGAS-STING-TBK1 signaling pathways. Studies show that NF-κB activity is significantly enhanced in macrophages infected with the ICP27 deletion mutant of HSV-1, suggesting that ICP27 can inhibit the cGAS-STING-TBK1 pathway (Christensen et al., 2016). Post-translational acetylation of the NF-κB subunit p65 plays an important role in regulating NF-κB activity. Multiple sites of p65 can be reversibly acetylated, with p300/CREB-binding protein (CBP) and p300/CBP-associated factor (PCAF) serving as the primary enzymes for p65 acetylation (Abraham, 2000). In contrast, histone deacetylase 3 (HDAC3) mediates p65 deacetylation (Chen and Greene, 2003). This process is inhibited by the death domain-associated protein (Daxx), a regulator of cell survival, apoptosis, gene expression, and the cell cycle (Park et al., 2007). SUMOylation of Daxx controls its nuclear anchoring and nucleocytoplasmic localization, inhibiting p65 acetylation and thus suppressing NF-κB transcriptional activity (Shih et al., 2007). Additionally, HSV-1 ICP27 may interfere with Daxx SUMOylation through spatial hindrance, enhancing the interaction between Daxx and p65, further suppressing p65 acetylation and inhibiting NF-κB signaling (Kim et al., 2017).

HSV-1 ICP0 targets and degrades host SUMOylated proteins, such as PMLand Sp100, through its E3 ligase activity, thereby inhibiting the recruitment of viral restriction factors (Everett et al., 2008). Furthermore, ICP0 disrupts the interaction between the E3 ligase protein inhibitor of activated STAT1 (PIAS1) and PML, preventing PIAS1 from localizing to the site of HSV-1 infection and replication within the host cell nucleus, which ensures efficient viral lysis and replication (Brown et al., 2016). In addition to these known SUMOylated targets, HSV-1 infection also alters the SUMOylation of various intracellular proteins. For example, Elizabeth Sloan and colleagues identified 124 intracellular SUMOylated proteins whose levels were reduced in response to ICP0 infection, including Zinc Finger and BTB Domain Containing 10 (ZBTB10), Zinc Finger and BTB Domain Containing 38 (ZBTB38), Moloney Leukemia Virus 10 Protein (MORC3), NAC Alpha Subunit 1 (NACC1), Ben Domain Containing 3 (BEND3), and Methyl-CpG Binding Domain Protein 1 (MBD1) (Sloan et al., 2015).

### 3.6 O-GlcNAc modifications in HSV-1 infection

Recent studies have demonstrated that HSV-1 infection enhances the host hexosamine biosynthesis pathway (HBP), promoting O-Linked N-Acetylglucosamine (O-GlcNAc) modifications and activating the STING signaling pathway, particularly through the succinylation of STING at Thr229 and K63-linked ubiquitination. This activation leads to an antiviral immune response. A mutation at Thr229 inactivates STING and reduces IFN production. Conversely, inhibition of O-GlcNAcylation using the drug6-Diazo-5-oxo-L-nor-Leucine (DON) significantly impairs the host’s ability to clear HSV-1, exacerbating infection and tissue damage. These findings highlight the crucial role of O-GlcNAc modification in antiviral innate immune responses (Li et al., 2024).

### 3.7 Citrullination modifications in HSV-1 infection

Protein citrullination involves the enzyme-catalyzed conversion of the imidazole group in arginine residues to a carbonyl group, resulting in the formation of citrulline, a non-genetically encoded amino acid. During HSV-1 infection, the virus activates protein arginine deiminase (PAD) enzymes (PAD2, PAD3, and PAD4), promoting hypercitrullination of interferon-induced proteins such as IFIT1 and IFIT2, thereby impairing interferon production. This citrullination represents a mechanism by which HSV-1 evades the immune response (Pasquero et al., 2023).

### 3.8 Crosstalk between PTMs in HSV-1 infection

PTMs are prevalent in virus-host interactions, with crosstalk frequently occurring between different PTMs (Beltrao et al., 2013). This crosstalk can be categorized into two mechanisms. The first group involves different modifications that interact by regulating the same substrate protein. In this context, modifications at specific amino acids can affect other modifications at the same site or nearby residues, synergistically regulating the function of the modified protein (Csizmok and Forman-Kay, 2018). Another type involves multiple enzymes with activating or inhibitory functions sequentially using each other as substrates, altering enzymatic activity through the addition or removal of modifications. This process forms positive regulatory or feedback loops to exert their functions. In PTM crosstalk, interactions between phosphorylation, ubiquitination, and SUMOylation have been the most commonly observed types of crosstalk (Garza-Domínguez and Torres-Quiroz, 2022; Hervás et al., 2020).

As previously discussed, HSV-1 ICP0 degrades SUMOylated proteins within PML nuclear bodies, thereby eliminating their antiviral activity. Concurrently, the host kinase Casein Kinase 1 (CK1) phosphorylates ICP0 at Thr67, enhancing its interaction with key proteins involved in the DNA damage response, such as RNF8 (Chaurushiya et al., 2012). Furthermore, Chk2 kinase promotes the phosphorylation of ICP0 at SUMO-interacting regions, which increases ICP0’s interaction with SUMOylated proteins and enhances the activity of SUMO-targeted ubiquitin ligase (STUbL) (Hembram et al., 2020). Additionally, the Us3 protein inhibits TRAF6-mediated ubiquitination of LAT, disrupting TCR signaling and preventing LAT phosphorylation, thereby suppressing T-cell activation and contributing to HSV-1 immune evasion (Yang et al., 2015). The HSV-1 UL9 protein interacts with nuclear factor B42 (NFB42), promoting viral replication. Co-expression of NFB42 and UL9 in 293T cells significantly reduces UL9 protein levels, which can be restored by the proteasome inhibitor MG132. This interaction, dependent on phosphorylation, suggests that NFB42 mediates UL9 polyubiquitination and subsequent proteasomal degradation (Eom and Lehman, 2003). These examples illustrate how viral proteins coordinate multiple PTMs, including ubiquitination, SUMOylation, and phosphorylation, to suppress host antiviral immune responses.

In addition to these inter-modification relationships, cascading effects between single types of PTMs have been reported. For example, as aforementioned, the HSV-1 UL37 protein functions as a deamidase and interacts with the host RIG-I, facilitating deamidation at the N495 site. This modified RIG-I can subsequently interact with another deamidase, PPAT leading to further deamidation at the N549 site. This process ultimately prevents RIG-I from recognizing the virus (Huang et al., 2021; Zhao et al., 2016).

## 4 Discussion

HSV-1 infection is a widespread phenomenon. Once a host undergoes primary infection, HSV-1 establishes a latent infection in the nervous system, which can persist for an extended period. HSV-1 promotes its survival by evading the host innate immunity through a variety of immune evasion strategies. Among these, the post-translational modification of proteins is one of the major ways which HSV-1 antagonizes the host antiviral innate immune response (Cakir et al., 2021). Therefore, studying the mechanisms related to the post-translational modification of HSV-1 viral proteins offers novel insights into the molecular mechanisms underlying HSV-1 infection latency and provides a theoretical foundation for the design of more effective therapeutic drugs and strategies.

Currently, drug development strategies targeting HSV-1 primarily center on host cell factors involved in viral replication. These drugs are known as host-targeted antiviral drugs (HTAs) (Su et al., 2024). Research is also in progress to develop drugs that target the viral PTMs during HSV-1 infection or those that influence host cell PTMs. For instance, inhibitors that target PADs can inhibit the citrullination of interferon-induced protein IFIT1 and IFIT2. This inhibition reverses HSV-1 immune evasion and effectively suppresses the infection (Pasquero et al., 2023). Additionally, NLRC4 has been shown to promote the interaction between TBK1 and the E3 ubiquitin ligase Casitas B-Lineage Lymphoma (CBL), enhancing K63-linked polyubiquitination of TBK1 Consequently, this upregulates the cGAS-STING signaling pathway and boosts antiviral innate immunity (Zhang et al., 2023).

In conclusion, HSV-1 viral proteins utilize PTMs, particularly phosphorylation and ubiquitination, to regulate immune evasion as a key strategy to counteract the host immune system. However, there are still relatively few targeted drugs aimed at viral protein PTMs. Therefore, elucidating the mechanisms of host-HSV-1 protein interactions and the PTMs involved in immune evasion will be crucial for guiding future immune therapies and drug development strategies targeting HSV-1. In the long run, this line of research holds the potential to yield more effective approaches for curbing HSV-1 infection and transmission within the population, thereby enhancing public health and reducing the burden associated with HSV-1-related diseases.
